# Resilience and Recovery in the Informal Economy: Social Networks, Social Protection, and Adaptive Strategies Among Post‐COVID Workers in Bangladesh

**DOI:** 10.1002/puh2.70295

**Published:** 2026-06-12

**Authors:** Md. Abdullah Al Mamun, Khandakar Farid Uddin, Md. Shawan Uddin, A. N. M. Jahangir Kabir, Md. Nazirul Islam Sarker

**Affiliations:** ^1^ CRSF Research Department of Folklore and Social Development Studies, Faculty of Social Science University of Rajshahi Rajshahi Bangladesh; ^2^ School of Social Sciences Western Sydney University Sydney New South Wales Australia; ^3^ Economics Discipline, School of Business University of Technology (UTS) Sydney New South Wales Australia; ^4^ Department of Management Studies, Faculty of Business Studies University of Rajshahi Rajshahi Bangladesh; ^5^ School of Public Administration Xi'an University of Finance and Economics Xi'an China; ^6^ Miyan Research Institute International University of Business Agriculture and Technology Dhaka Bangladesh

**Keywords:** Bangladesh, COVID‐19, informal economy, poverty alleviation, social protection

## Abstract

The informal economy is vital in developing countries by providing income opportunities to marginalized populations. The COVID‐19 pandemic intensified existing vulnerabilities, causing severe income loss and heightened insecurity. The study aims to investigate the impact of COVID‐19 on informal workers’ income and livelihoods, explore their adaptation strategies, and assess the role of social networks and social protection in enhancing resilience. Using a pragmatist paradigm and an exploratory sequential mixed methods design, the study collected qualitative data through 12 in‐depth interviews and quantitative data from 500 survey respondents, selected via purposive and snowball sampling. Thematic analysis and regression models were employed to interpret the findings. Results show that over 50% of informal workers experienced income decline, with many adopting coping strategies such as taking on additional jobs, reducing expenses, and seeking informal credit. Only 10% accessed formal social protection. Strong social networks proved vital, offering financial and emotional support. Regression analysis confirmed that income loss significantly increased financial insecurity (*β* = −0.45, *p* < 0.01), whereas social networks (*β* = 0.40, *p* < 0.01) and social protection (*β* = 0.35, *p* < 0.01) positively influenced resilience. The study also found that respondents with access to informal credit networks or community organizations fared significantly better than others. The study highlights the urgent need for inclusive social protection, vocational training, and community‐based support to enhance the informal sector's resilience. Findings offer actionable insights for policymakers and development actors working toward sustainable recovery.

## Graphical abstract

Post‐COVID informal workers in Bangladesh experienced about 50% income loss, driving financial insecurity with public‐health consequences. Resilience was strengthened by income diversification and strong social networks, whereas only about 10% accessed formal social protection—highlighting the need for inclusive, community‐linked safety nets to support recovery and health resilience.

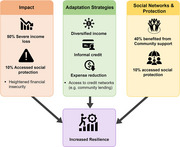

## Introduction

According to the 17th International Conference on Labor Statistics (ICLS), informal employment includes any jobs that do not fall under either formal or informal sector. It includes both formal and informal jobs, as well as those held by households or businesses. This includes jobs that are not part a regulated structure, and where the workers don't pay tax on their earnings [1]. It is possible to work both in formal and informal jobs at the same. Because informal workers lack access to social protection, education, and credit, they are more likely to earn less than their formal counterparts [2].

Informal economies are the main source of employment and economic activity in many developing countries. The informal sector in Bangladesh includes a wide range of activities that are not regulated or registered. Street vending and small‐scale production are included [3, 4]. Workers in the informal economy face many challenges. These include a lack of legal employment, job security, and social protection. They are more vulnerable to poverty and economic shocks [5]. COVID‐19 increased this vulnerability and has disrupted the livelihoods of unorganized workers. Many workers in the unorganized sectors have lost their jobs and income due to the restrictions and lockdowns implemented to stop the virus spreading [6]. The workers struggle to meet their basic needs because they do not have enough savings and social safety nets [7]. Bangladesh's informal sector has been particularly affected. Researchers have found that workers from the informal sector are more vulnerable and have suffered significant income losses [4, 8, 9, 10]. When faced with these obstacles, they have shown remarkable resilience. They have adopted a variety of strategies to adapt and stay afloat. These strategies show that the sector can recover with the right support from policymakers.

Recent studies have shown the informal economy's resilience and adaptability in times of crisis. Roever and Skinner [11] show how informal workers use innovative coping techniques to cope with systemic stress. These include diversifying income sources, using social capital, and adapting online marketplaces. Adger [12] and Folke [13] conceptualize resilience in terms of adaptive capacity, whereby social networks, community‐based support systems, flexible livelihoods, and other factors enable informal actors to absorb and recover after economic shocks. In countries such as India, Kenya, and Brazil, research during the pandemic shows that strong informal networks can often replace missing state support [14]. There are limitations to these coping mechanisms. Without formal social protection programs, such as food security, cash transfers, or health insurance, the capacity of informal workers to recover is fragile. Hossain [15], Swarna et al. [16], and [16, 17, 18] have highlighted the severe disruptions that informal workers experienced during COVID‐19. The lockdowns resulted in widespread income losses for workers, particularly those on daily wages who had no savings or a digital financial system. Although community‐based assistance and non‐government aid played a key role in the immediate response, structural gaps in state social protection remain.

Despite the critical role of the informal economy in providing employment and income opportunities in developing countries like Bangladesh, important gaps remain in understanding how informal workers experienced, managed, and recovered from the shocks of the COVID‐19 pandemic. Previous studies have highlighted the vulnerabilities faced by informal workers, including limited social protection, insecure employment, and restricted access to credit [4, 16, 19, 20, 21]. However, less attention has been given to how these workers adapted over time, how social networks and social protection shaped resilience, and how these processes varied across underrepresented regional contexts [8, 10, 22, 23]. In particular, northern Bangladesh has received limited scholarly attention despite its substantial concentration of informal workers and distinct livelihood vulnerabilities. By focusing on this region and examining both immediate disruptions and longer term recovery dynamics, this study addresses an important empirical and policy gap in the literature. Therefore, this study focuses on the following key questions:

1. How has the COVID‐19 pandemic affected the income and livelihoods of informal workers in Bangladesh?
2. What adaptation strategies have informal workers employed to cope with the economic impact of the pandemic?
3. How do social networks influence the resilience of informal workers during the pandemic?
4. What role can social protection measures play in enhancing the resilience and recovery of informal workers?

By addressing these questions, the study examines how informal workers in Bangladesh experienced disruption, adaptation, and recovery in the post‐pandemic period, with particular attention to resilience and livelihood adjustment. Understanding these processes provides policy‐relevant evidence for governments and development actors seeking to strengthen poverty alleviation, inclusive social protection, and informal sector recovery in developing‐country contexts.

This study makes several contributions to the literature on informal economies and social protection. First, it integrates resilience theory and dual economy theory to provide a more comprehensive explanation of vulnerability, adaptation, and recovery among informal workers. Second, by focusing on northern Bangladesh, the study adds sub‐national evidence from an underrepresented regional context within the broader literature on informal labor and post‐pandemic recovery. Third, the study empirically examines the complementary roles of formal social protection and informal social support mechanisms in shaping resilience outcomes. Fourth, the findings reveal that informal workers often relied on hybrid coping strategies that combined income diversification, expenditure reduction, informal credit, and community‐based support. Finally, the study offers policy‐relevant insights for the design of more inclusive and context‐sensitive social protection and recovery programs in Bangladesh and comparable developing‐country settings.

The remainder of the article is structured as follows: Section 2 reviews the literature and theoretical framework; Section 3 outlines the methodology; Section 4 presents findings; Section 5 discusses implications; and Section 6 concludes with policy recommendations.

## Literature Review, Theoretical Background, and Hypotheses Development

### The Informal Economy in Global and National Contexts

The informal economy is a major part of the global economy, particularly in developing countries. ILO [3] defines informal economy as activities that are not governed by legal or practical agreements. These activities provide employment and income for millions of people who would otherwise be unemployed. This sector is marked by poor working conditions, lack of social protection, and limited access to markets and resources [5]. In Bangladesh, the informal economy is crucial for the livelihood of many people. According to research, a large part of the population is employed in informal jobs that contribute significantly to the GDP of Bangladesh [24]. In spite of its importance, the informal sector is not adequately studied and receives inadequate policy support. Its workers are, therefore, left vulnerable [19]. The COVID‐19 pandemic has had a serious impact on the world economy. The COVID‐19 pandemic caused major disruptions to the informal sector. Pandemic‐induced restrictions led to a loss of income and livelihoods for informal workers [7].

Recent research has examined the effects of COVID‐19 on informal workers in Bangladesh [9, 10, 16, 25], yet important gaps remain given the diversity and dynamism of the informal economy. First, although several studies focus on the immediate shocks of the pandemic, less is known about the sustainability of adaptation strategies and the processes of longer term livelihood recovery among informal workers [26]. Second, existing literature often emphasizes national or aggregate patterns, giving insufficient attention to sub‐national variation and occupational diversity in underrepresented regions such as northern Bangladesh. Third, although the importance of social protection is widely acknowledged, limited empirical work has examined how formal support mechanisms interact with informal social networks to shape resilience outcomes [2]. In addition, many previous studies rely on a single‐method design, leaving room for more integrated mixed‐methods evidence [27]. These gaps justify the present study's focus on informal workers in northern Bangladesh and its attention to both immediate disruption and longer term recovery.

To address these gaps, this study employs an exploratory sequential mixed‐methods approach to examine informal workers in northern Bangladesh. It investigates both the immediate and longer term impacts of COVID‐19 on income, livelihoods, adaptation strategies, social networks, and social protection, thereby generating regionally grounded evidence for strengthening inclusive recovery, poverty alleviation, and social protection policy.

### COVID‐19 and Informal Livelihood Disruptions

The COVID‐19 pandemic deeply disrupted the informal economy worldwide, particularly through income losses, increased food insecurity, and reduced access to healthcare [3, 28]. Informal workers, without contracts or social safety nets, were the first to lose employment and the last to receive assistance. In Bangladesh, 98% of respondents reported that they had seen a decrease in their monthly income during the lockdown. This average monthly drop was BDT 6800 [29]. Many informal workers lacked the coping mechanisms or institutional support to increase their vulnerability [4]. The pandemic brought to light the vulnerability and precariousness that informal workers face, as well as the need for them to be integrated into social protection programs.

### Resilience and Adaptation in the Informal Sector

According to reports, informal workers were able to adapt themselves to pandemics through diversifying their sources of income, utilizing digital platforms and relying on social networks [3, 11, 30]. This adaptation strategy shows how flexible the system is, even without the state's intervention. Bangladesh's recovery and survival were dependent on community‐based networks [31].

### The Role of Social Protection

Social protection programs such as food and cash assistance, as well as subsidized healthcare, were crucial in reducing adverse effects from crises such as COVID‐19. Informal workers who do not have formal contracts or registrations often are excluded. Social networks are known to be a major support for informal workers. The lack of formal protection makes many vulnerable to hardships over the long term [18]. It is important to have a balance between the informal social support system and more institutionalized protection mechanisms that are inclusive [1].

### Theoretical Framework

To gain insight into the dynamics and challenges of the informal economy, it is best to analyze this through a variety of theoretical lenses. The informal economy can be viewed through the lens of resilience. This research is relevant to the dual economy theory and resilience theory. This study analyzes the dynamics of informality using two key theoretical frameworks:

#### Dual Economy Theory

Lewis [32] and Hart [33] describe the division of economies in formal and informal segments. The formal sector is highly regulated and has a large capital intensity, whereas the unregulated informal sector is high in labor intensity. In Bangladesh, this dualism reflects structural inequality. Informal work is often the “last option” for survival [5]. According to dual economy theory, economies in developing countries are divided into formal and informal sectors. The formal industry is regulated and has significant capital investments and stable employment. In contrast, the unregulated informal sector has unregulated, precarious employment with little capital. The informal sector, despite its vulnerability, is essential for employment and livelihoods where formal jobs are rare [34]. Bangladesh's informal economy makes up a significant part of the labor market. This shows the dualistic nature of the economy.

#### Resilience Theory

In the past, resilience theory has been used widely to explain how social systems recover from shocks and recover [13]. In informal work contexts, resilience is determined primarily by the resources, adaptive capability, and institutional support. In the context of the informal economy, resilience refers to the ability to adapt and thrive in the face of economic disruptions, such as COVID‐19 [13]. Adger [12] explains how resilience is affected by many factors such as social networks and resources. Understanding these factors can help you create policies that will enhance resilience in Bangladesh's unorganized economy.

**FIGURE 1:**
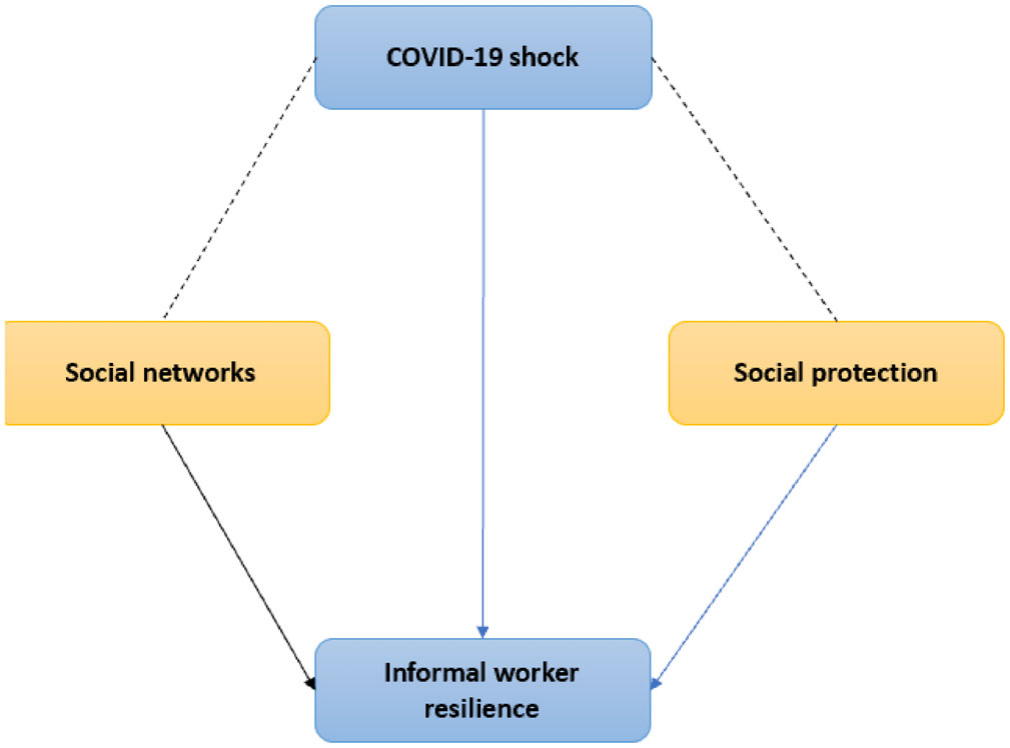
Conceptual model linking COVID‐19 shock, social networks, social protection, and informal worker resilience.

These frameworks provide a conceptual lens to understand the vulnerability and adaptation potential of Bangladesh's unorganized workforce as they react to the pandemic (Figure 1).

The theoretical framework underpinning this study moves beyond a simple application of the dual economy and resilience theories. First, this research demonstrates that resilience in the informal sector is not solely an individual or household attribute, but an emergent property of the dynamic interplay between formal state systems and informal social networks. Second, by empirically documenting how informal workers navigate gaps in state protection through adaptive use of kinship, neighborhood, and community resources, this study contributes a nuanced perspective to resilience theory: It highlights the importance of “institutional bricolage,” where actors combine and repurpose available support mechanisms.

Third, the findings challenge the rigid dualism of Lewis's [32] framework by illustrating the hybrid nature of informal workers’ coping strategies, which blend formal, semi‐formal, and informal institutions—particularly visible in adopting digital financial services and cross‐sector partnerships during COVID‐19. Finally, the proposed synthesis calls for an “adaptive systems” lens: post‐pandemic resilience in the informal economy is best understood as a function of policy flexibility, multi‐level institutional collaboration, and social capital activation. This novel approach offers a more dynamic and policy‐relevant theoretical foundation for research on vulnerability, adaptation, and social protection in the Global South.

More specifically, the combined theoretical framework helps explain why resilience in the informal economy is often produced through hybrid arrangements rather than through either market participation or state support alone. In the Bangladeshi context, informal workers navigated crisis through an interdependent mix of household adjustment, community‐based reciprocity, informal credit, and limited forms of formal assistance. This perspective also helps illuminate threshold effects in social support, whereby resilience improved more substantially when informal support networks were reinforced by at least minimal institutional or programmatic protection.

Overall, dual economy theory explains the structural vulnerability of informal workers, whereas resilience theory clarifies the mechanisms through which they absorb, adapt to, and recover from crisis‐related shocks. These complementary perspectives provide the conceptual basis for the research questions and hypotheses developed in this study.

### Hypotheses Development

On the basis of the reviewed literature and the integrated theoretical lens of dual economy theory and resilience theory, the study is guided by four hypotheses concerning income shock, adaptation, social networks, and social protection in the informal economy. The COVID‐19 epidemic in Bangladesh affected livelihoods and income for informal workers. The lack of social protection and precarious nature of informal employment in Bangladesh has led to the following hypothesis:

H 1 The COVID‐19 pandemic has led to a significant decrease in the income of informal workers in Bangladesh.

Informal workers employ a wide range of strategies to cope with economic shocks. These strategies are crucial for their resilience in crisis situations and recovery. It is hypothesized that

H 2 Informal workers in Bangladesh have employed diverse adaptation strategies to cope with the economic impact of the COVID‐19 pandemic.

Social networks can be a vital source of support and resources during a crisis. Informal workers often rely on these networks for information and emotional support. It is hypothesized that

H 3 Strong social networks positively influence the resilience of informal workers in Bangladesh during the COVID‐19 pandemic.

Lack of social protection measures increases the vulnerability of informal workers. Social protection can reduce the negative effects of economic shocks and increase resilience. It is hypothesized that

H 4 The implementation of social protection measures positively impacts the resilience and recovery of informal workers in Bangladesh.

**FIGURE 2:**
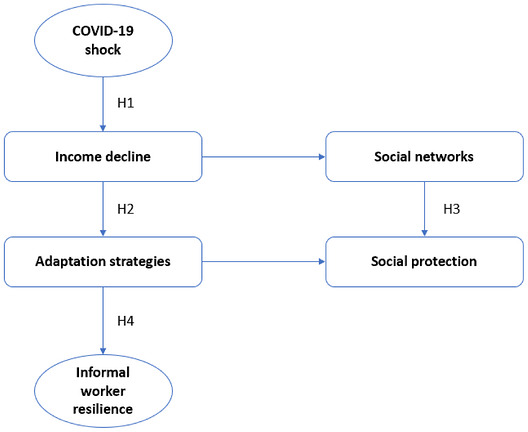
Hypothesized relationships among major factors.

Figure 2 maps the core hypotheses of this study (H1–H4) to ensure conceptual consistency and clarity. It gives a visual overview of the expected interactions between the pathways.

## Methodology

### Research Design

This study employs a pragmatic research paradigm that integrates both qualitative and quantitative methodologies in order to gain a complete understanding of Bangladesh's informal economic system [35]. An exploratory sequential mix‐methods approach was adopted; first, qualitative data were gathered during COVID‐19 to explore experiences, vulnerabilities, and coping strategies of informal workers; second, emergent themes led to designing of structured questionnaires to collect quantitative data that validated and extended initial findings.

### Feature of the Study Area

**FIGURE 3:**
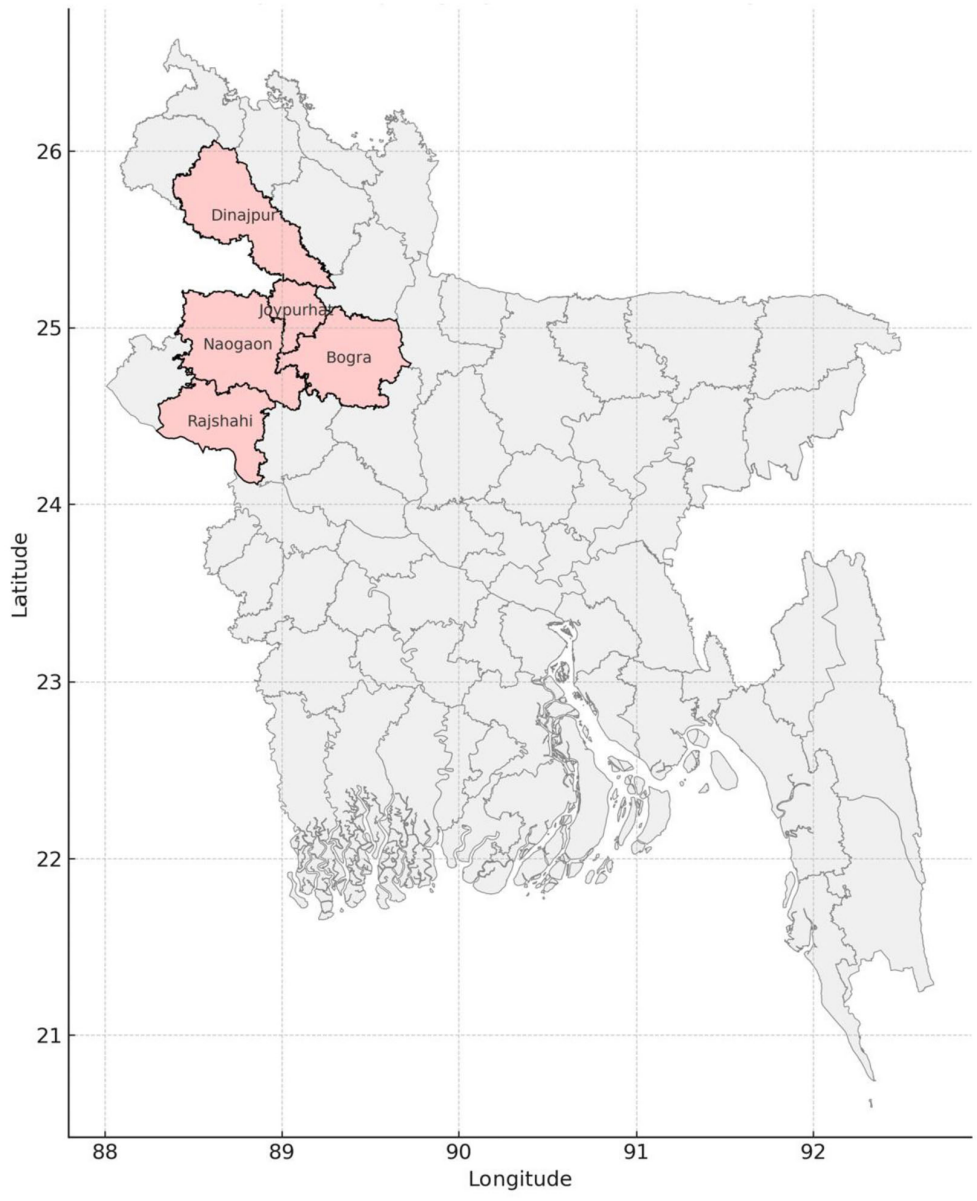
Study areas (highlighted districts) in Bangladesh.

The study took place in selected districts in Bangladesh's northern zone, such as Rajshahi (Figure 3), Nogoan, Joypurhat, and Bogura, chosen based on their high concentration of informal workers specializing in street vending, rickshaw‐pulling, domestic service provision, and day labor activities—giving an accurate representation of informal economy outside metropolitan centers.

### Sampling, Sample Size, and Data Collection

The study participants were selected using a multistage sampling technique. In the first phase, districts were selected on purpose based upon the presence of informal economic activity. In the second phase, in each district selected, informal workers were identified using a combination purposive and snowball sampling methods. This was done to ensure that there was a diversity of occupations, experiences, and backgrounds [36]. The researchers used a purposive sample technique to conduct IDIs in the qualitative phase. The researchers used a personal contact approach, similar to the one described by White et al. [37]. Fugard and Potts [38] note that sample size is relative in qualitative research and cannot be calculated or determined beforehand. In certain homogeneous case sampling or essential cases, a sample of 10 can be considered sufficient [8]. In order to uncover the nuanced strategies and experiences of informal workers, 12 IDIs were used. A semi‐structured interview guide was developed to ensure consistency while allowing flexibility to explore emerging themes [39]. The interview guide covered livelihood disruption, coping strategies, access to support, and recovery experiences and can be shared in anonymized form upon reasonable request. The interviews were audio‐recorded, transcribed, and translated into English for analysis. Table 1 illustrates the profiles of the field research interview participants.

**TABLE 1:** Demographic profile of the respondents.

Respondents code	Gender	Age (years)	Occupation	Education level	Interview date
1	M	22	Street vendor	Primary education	18.10.23; 2.00 p.m.
2	F	28	Domestic worker	No formal education	19.10.23; 4.00 p.m.
3	M	39	Rickshaw puller	Primary education	20.10.23; 2.30 p.m.
4	M	43	Day laborer	Primary education	22.10.23; 3.30 p.m.
5	M	37	Street vendor	Secondary education	24.10.23; 2.30 p.m.
6	F	48	Domestic worker	Primary education	26.10.23; 4.30 p.m.
7	F	32	Street vendor	No formal education	27.10.23; 12.30 p.m.
8	M	27	Rickshaw puller	Primary education	29.10.23; 2.30 p.m.
9	M	29	Rickshaw puller	Primary education	02.11.23; 12.30 p.m.
10	F	33	Day laborer	No formal education	03.11.23; 1.00 p.m.
11	M	40	Day laborer	Primary education	05.11.23; 10.00 a.m.
12	M	28	Day laborer	Primary education	05.11.23; 2.30 p.m.

Table 1 provides details of individual respondents, including their gender, age, occupation, education level, and interview date and time. The sample consists of seven males and five females. The ages range from 22 to 48 years. Most respondents are in their late 20s to early 40s. The occupations include street vendors, domestic workers, rickshaw pullers, and day laborers. The most common occupation among the respondents is day laborer (four respondents). The education levels vary, with the majority having primary education. A few respondents have no formal education, and only one has secondary education. The interviews were conducted from 18.10.23 to 05.11.23, with varying times. The data present a diverse sample in terms of gender, age, and occupation, but a relatively low level of education. Most respondents have primary education, and the most common occupations are day laborers and rickshaw pullers. The data also show the scheduling and execution of interviews over several weeks.

On the other hand, for the quantitative portion of this study, the researchers utilized combination of purposive and snowball sampling techniques to conduct the survey. Structured questionnaires were distributed to respondents using face‐to‐face interviews, resulting in 510 responses, of which 500 were usable. This sample size meets the requirement of optimum sample size, assuming a 99% confidence level, a standard deviation of 0.5, and a ±1% error margin. Quantitative data were collected through structured surveys administered to a larger sample of informal workers. The survey questionnaires were developed based on the findings from the qualitative phase and existing literature. It included questions on demographic characteristics, income changes, adaptation strategies, social networks, and access to social protection [27]. A structured survey instrument and the semi‐structured interview guide were used to ensure consistency across respondents; these materials, together with the variable descriptions/codebook used for analysis, are available from the corresponding author upon reasonable request for academic purposes. The survey was administered using face‐to‐face interviews, ensuring a high response rate and data accuracy. Table 2 illustrates the respondents’ profiles of the survey. All interviews and surveys were conducted voluntarily with prior oral informed consent, and respondent confidentiality and anonymity were protected throughout the study.

**TABLE 2:** Descriptive analysis of demographic data of the respondents.

Demographic data	Frequency (*N* = 500)	Percentage
Gender		
Male	325	65
Female	175	35
Age		
18–25 years old	87	17.31
26–35 years old	96	19.23
36–45 years old	154	30.76
46–55 years old	144	28.85
56 and above years	19	03.85
Occupation		
Day laborer	103	20.60
Street vendor	114	22.80
Rickshaw puller	178	35.60
Domestic worker	105	21.00
Education level		
No formal education	274	54.80
Primary education	218	43.60
Secondary education	6	1.20
Higher secondary education	2	040
Impact of COVID‐19 on income		
Average monthly income before COVID‐19 (in BDT)		
Less than 5000	76	15.20
5000–10,000	92	18.40
10,001–15,000	147	29.40
More than 15,000	185	37.00
Average monthly income during COVID‐19 (in BDT)		
Less than 5000	325	65.00
5000–10,000	160	32.00
10,001–15,000	12	2.40
More than 15,000	3	0.60

Table 2 provides demographic and economic impact data for a sample of 500 individuals. The table breaks down the data into several categories: gender, age, occupation, education level, and the impact of COVID‐19 on income. The sample is predominantly male, with males constituting 65% of the sample and females 35%. The largest age group is 36–45 years old (30.76%), followed by 46–55 years old (28.85%). The smallest group is 56 and above years old (3.85%). Rickshaw pullers form the largest occupational group (35.60%), followed by street vendors (22.80%), domestic workers (21.00%), and day laborers (20.60%). A significant majority of the sample has no formal education (54.80%), with primary education being the next largest group (43.60%). Very few have secondary (1.20%) or higher secondary education (0.40%). There has been a significant shift in income levels due to COVID‐19. Before the pandemic, a majority had incomes above 10,000 BDT (66.40%). During the pandemic, 65% of individuals have an income of less than 5000 BDT. The percentage of individuals earning more than 10,000 BDT has drastically reduced from 66.40% to 3%. Overall, the data indicate a predominantly male, middle‐aged, low‐educated, and low‐income demographic. The impact of COVID‐19 has been severe, with a drastic reduction in income levels for the majority of the sample.

### Data Analysis

Qualitative data from IDIs were analyzed using thematic analysis, following Braun and Clarke's [40] six‐step framework: (1) familiarization with data, (2) generating initial codes, (3) searching for themes, (4) reviewing themes, (5) defining and naming themes, and (6) producing the final report. NVivo software was used to facilitate coding and thematic mapping. Themes were identified inductively and refined through iterative analysis.

Quantitative data were analyzed using IBM SPSS Statistics (version 25). Descriptive statistics (mean, frequency, percentages) were used to summarize respondent characteristics and pandemic‐related income changes. Inferential statistics—including Pearson's correlation and regression analysis—were employed to test hypotheses concerning the relationships between income decline, financial insecurity, resilience, social networks, and social protection. To ensure measurement validity and reliability, Cronbach's alpha and confirmatory factor analysis (CFA) were used. Goodness‐of‐fit indices such as CFI, TLI, and RMSEA were assessed, following guidelines by Hair et al. [41]. The integration of thematic evidence from the qualitative phase with statistical findings from the quantitative phase strengthened methodological triangulation and enhanced the robustness of interpretation.

## Results and Discussion

### Findings of the Qualitative Phase

The qualitative phase revealed three central themes: income disruption, coping mechanisms, and social support networks. Informal workers consistently described a substantial decline in income due to the COVID‐19 lockdowns, leading to heightened financial insecurity. Participants reported difficulty in meeting basic needs, including food, rent, and healthcare. Regarding these respondents 1, 2, and 3 mentioned,

The COVID‐19 pandemic led to a substantial decline our income, increasing our financial insecurity and vulnerability to poverty.

To cope, many participants diversified their income sources by engaging in new activities such as online sales, home‐based work, or small‐scale farming. Others reduced non‐essential expenses and negotiated temporary rent relief. Respondents 2, 5, 8, 10, and 11 noted:

We had to cut back on buying anything that isn't absolutely necessary. Even when it comes to rent, we have talked to our landlord to reduce it, at least temporarily. Medical expenses are kept to a minimum, only going to the doctor if it's really urgent.

Respondents 4, 7, 8, and 10 highlighted the crucial role of family, friends, and community organizations in providing financial, material, and emotional support. Informal workers demonstrated resilience by employing various adaptation strategies, including income diversification, expense reduction, and reliance on social networks. They said during the interview:

We wouldn't have been able to cope without the help of our friends and family. We've had support from them both financially and emotionally. Local community groups have also provided us with assistance. To get by, we have cut back on expenses and relied heavily on social networks.

Social networks that were strong played an important role in providing resources and support to the informal workers. This increased their resilience during this crisis. The lack of social safety measures also left the informal workers vulnerable. Social protection programs are crucial to enhancing resilience and recovery. In this regard, respondents 1, 5, 9, and 12 stated that

Having a solid social network was a crucial factor in our resilience. Families and friends have helped in many ways, including financial assistance or emotional encouragement. This has been crucial for me to get through this difficult period.

Respondents 2, 6, and 11 mentioned that

Our social networks have been extremely important during this period. Our friends and family provided us with emotional and financial assistance, which helped us remain resilient despite our challenges.

Participants of the IDIs in this study reported that the COVID‐19 epidemic significantly reduced the income of Bangladeshi informal workers, increasing their financial vulnerability. As a response, they implemented strategies like reducing non‐essential expenses, negotiating a rent reduction, and minimizing healthcare costs. Support from social networks such as family, friends, and community groups was crucial in providing emotional, financial, and material assistance. The resilience of informal workers was a result of their income diversification and reliance on the social network. The lack of formal protection left them vulnerable. This highlights the need to implement social protection programs in order to increase their resilience and support recovery. These qualitative insights informed and contextualized the subsequent quantitative analysis by identifying the principal dimensions of vulnerability, coping, and resilience examined in the survey phase.

### Findings of the Quantitative Phase

To validate these qualitative findings, survey responses from 500 informal workers were analyzed using structural equation modeling. Construct validity and internal consistency were confirmed, as shown in Table 3.

**TABLE 3:** Validity and reliability of constructs.

Construct	Cronbach's alpha	Composite reliability	CFI	TLI	RMSEA
Income decline	0.850	0.840	0.95	0.94	0.05
Financial insecurity	0.870	0.880	0.95	0.94	0.05
Resilience	0.830	0.820	0.95	0.94	0.05
Social networks	0.890	0.870	0.95	0.94	0.05
Social protection	0.800	0.790	0.95	0.94	0.05

Table 3 summarizes reliability and validity metrics of constructs used in this study. This is necessary to ensure the consistency and strength collected data. Cronbach's alpha measures the internal consistency. The item within a construct represents the same concept. Values above 0.7 are considered acceptable [42]. Cronbach's alpha is between 0.800 and 0.890, for all constructs in this research. This indicates a solid level of internal consistency. Cronbach's alpha for social networks, for example, is 0.890. This shows that the construct being measured can be accurately measured. Composite reliability (CR) is a measure for overall construct reliability. It is similar to Cronbach's alpha but more suitable to CFA [41]. All constructs show CR values between 0.790 and 0.880, which indicate high reliability.

The Comparative Fit Index measures the model's suitability in comparison to a starting model. Values over 0.90 indicate a good fit [43]. CFI values are set at 0.95 for each construct. This shows that the model fits the data well. The Tucker–Lewis Index (also known as Non‐Normed Fit Index) compares model fit to a null‐model while taking into consideration model complexity. The TLI for all constructs is 0.94, which indicates good model fit. The Root Mean Square approximation (RMSEA), a measure of model fit to the population covariance matrices [44], is less than 0.08 and indicates a good approximation. RMSEA values below 0.05 are considered acceptable for all constructs. The measurement instruments in this study were both valid and reliable, and they provide a solid basis for data interpretation and analysis.

Table 4 shows the results of the hypothesis test, which examines the relationship between the key variables. The regression analysis is used to support each hypothesis. It evaluates the path coefficients, standard errors, *t* values, and corresponding *p* values. Overall, the quantitative results provide empirical support for the study hypotheses and statistically reinforce the key patterns identified in the qualitative phase.

**TABLE 4:** Result of the path coefficient.

Hypothesis	Path	*β*	SE	*t* value	*p* values
H1	COVID‐19 pandemic → Income decline	−0.50	0.079	6.32	<0.01
H2	Income loss → Financial insecurity	−0.45	0.076	5.89	<0.01
H3	Social networks → Resilience	0.40	0.072	5.52	<0.01
H4	Social protection → Resilience and recovery	0.35	0.069	5.10	<0.01

The path coefficient (*b* = −0.50; SE = 0.07, *t* value 6.32, *p* <0.01) shows a negative relationship between income decline and the COVID‐19 epidemic, which supports the hypothesis that this pandemic caused a substantial drop in income for informal workers. The path coefficient (*β* = −0.45, SE = 0.076, *t* value = 5.89, *p* < 0.01) demonstrates a significant negative association between income loss and financial insecurity, suggesting that reduced income heightened economic vulnerability. The path coefficient (*β* = 0.40, SE = 0.072, *t* value = 5.52, *p* < 0.01) indicates a positive relationship between social networks and resilience, suggesting that strong social ties contribute to enhanced resilience during crises. The path coefficient (*β* = 0.35, SE = 0.069, *t* value = 5.10, *p* < 0.01) shows a positive relationship between access to social protection and resilience/recovery, highlighting the importance of social safety nets in mitigating adverse effects of economic shocks.

Although women constitute 35% of the survey sample, the experiences of female informal workers often differed from those of their male counterparts in ways that reveal broader gendered and intersectional vulnerabilities. Women were more concentrated in lower‐paid and less secure occupations, such as domestic work and street vending, and reported greater barriers to accessing formal social protection and informal credit networks. Qualitative evidence also suggested that these disadvantages were compounded by low educational attainment, caregiving responsibilities, and social stigma in some cases. These findings add depth to the analysis of resilience by showing that recovery capacity in the informal economy is shaped not only by income loss, but also by intersecting social and structural disadvantages. This underscores the need for gender‐responsive and socially inclusive policy interventions, including targeted cash transfers, childcare support, and improved access to support services.

**FIGURE 4:**
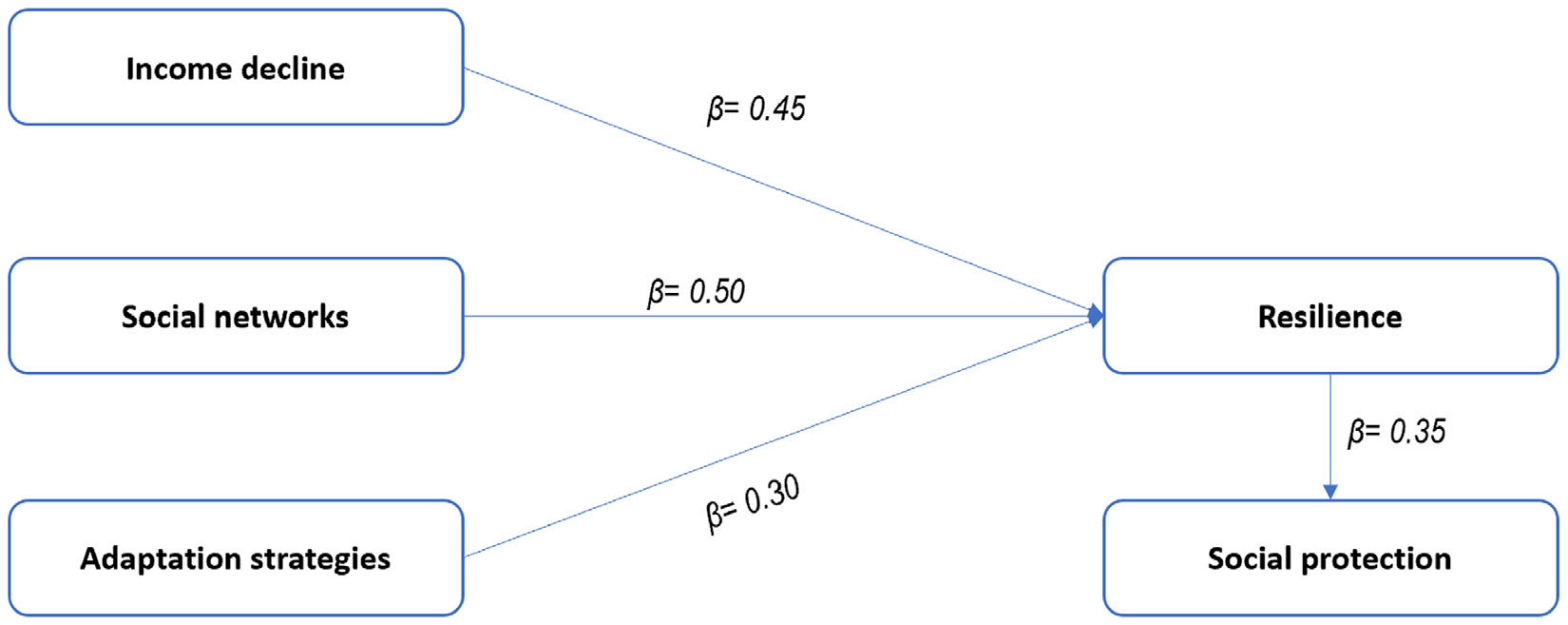
Path analysis of factors affecting informal worker resilience.

Figure 4 visually presents the results of the path analysis, illustrating the standardized coefficients for the relationships between key variables affecting informal worker resilience. This diagram highlights the positive effects of social networks and social protection and the negative impact of income decline on resilience outcomes.

### Integration of Qualitative and Quantitative Findings

The mixed‐methods synthesis shows that resilience and recovery in the informal economy were shaped by the interaction of formal and informal support mechanisms rather than by any single factor alone. Informal workers responded to the economic impacts of COVID‐19 through hybrid coping strategies, including income diversification, expenditure reduction, reliance on kinship and community networks, and, where available, access to social protection. These findings extend existing literature by showing that recovery was more feasible when informal social support was complemented by at least some form of institutional assistance, thereby underscoring the layered and interdependent nature of resilience in the informal economy. These strategies were vital in maintaining resilience during the crisis, although the absence of formal social protection often constrained their effectiveness.

#### Income Shock and Financial Insecurity

Both qualitative and quantitative results show a 50% average reduction in income, with significant consequences for food security, healthcare access, and rent payment. In the quantitative data analysis, the researchers found a substantial decline in the income of informal workers due to the COVID‐19 pandemic. On average, respondents reported a 50% reduction in their monthly income. The regression analysis confirmed that the pandemic had a significant negative impact on income (*p* < 0.01), supporting H1. The income loss led to increased financial insecurity and difficulties meeting basic needs such as food, housing, and healthcare. Informal workers employed various adaptation strategies to cope with the economic impact of the pandemic. The thematic analysis identified three main themes: diversification of income sources, reduction of expenses, and reliance on social networks. Many respondents took on additional jobs, such as selling goods online or engaging in agricultural activities, to supplement their income. Others cut down on non‐essential expenditures and negotiated lower rents with landlords. These findings support H2.

#### Adaptation and Coping Mechanisms

Respondents diversified income sources, reduced spending, and relied heavily on personal networks—strategies echoed in thematic and statistical analyses. Social networks played a crucial role in supporting informal workers during the crisis. Respondents reported receiving financial assistance, food, and emotional support from family, friends, and community organizations. Regression analysis showed that strong social networks positively influenced the resilience of informal workers (*p* < 0.01), supporting H3. Social networks provided a safety net that helped individuals navigate the economic challenges posed by the pandemic.

#### Role of Social Networks and Protection

Social networks emerged as the primary buffer against economic shock. However, formal social protection was largely absent, exacerbating structural vulnerability. The absence of formal social protection measures exacerbated the vulnerabilities of informal workers. The survey results indicated that only 10% of respondents had access to any form of social protection, such as cash transfers or food aid. Respondents expressed a strong need for government support to mitigate the adverse effects of economic shocks. The regression analysis revealed that access to social protection significantly enhanced the resilience and recovery of informal workers (*p* < 0.01), supporting H4.

Moving beyond descriptive reporting, this study identifies and explains the underlying mechanisms that shape resilience in the informal sector. The regression results and qualitative narratives converge to suggest that access to strong social networks and some forms of institutional support are not merely associated with but are critical enablers of adaptive capacity and recovery. This aligns with recent “adaptive systems” models in resilience theory, which posit that multi‐level institutional linkages and social capital jointly buffer shocks [13]. The findings further indicate a threshold effect: below a certain level of social or financial support, informal workers are unable to diversify or sustain coping strategies, resulting in persistent vulnerability. These causal insights contribute to theoretical debates on the hybrid, networked nature of resilience in precarious economies and emphasize the importance of integrated policy approaches. This triangulation provides empirical depth to resilience theory, showing that adaptive capacity in low‐income informal populations is relational, driven by access to networks and external support systems, rather than individual agency alone.

Overall, the findings move beyond descriptive accounts of pandemic hardship by identifying the mechanisms through which social networks, social protection, and social position jointly shaped resilience and recovery in the informal economy.

### Implications of the Study

The findings emphasize the need for policy interventions that not only enhance formal social protection coverage but also leverage the strengths of existing community networks to support informal workers’ resilience and recovery. Policies should be developed to fully incorporate these informal support networks into broader institutional frameworks for maximum impact. Empowering community groups and NGOs is also key for strengthening vulnerable groups’ resilience. Such networks may continue offering informal workers essential emotional, financial, and material assistance. The provision of affordable healthcare or subsidies for informal workers could improve their health and ease financial strains. Informing workers on financial management, budgeting, and saving will help them better manage their finances as well as reduce economic downturns. Specialized training programs for informal workers can help them adapt to changing economic conditions, diversify employment options, and decrease their reliance on a single income source.

The policy implications of this study extend beyond broad recommendations for expanded social protection. First, the findings align with and reinforce the objectives of Bangladesh's National Social Security Strategy (NSSS), which calls for more inclusive and adaptable safety nets targeting the most vulnerable, including informal workers. However, the limited access of informal workers to existing schemes, such as the Vulnerable Group Development (VGD) program and the Employment Generation Program for the Poorest (EGPP), reveals persistent gaps in operationalization [16]. Second, this research suggests that existing frameworks need to incorporate portable, contributory micro‐insurance and cash transfer schemes that leverage mobile banking and community organization partnerships, as piloted during COVID‐19 in parts of India. These operational models provide lessons for Bangladesh in reaching unregistered informal workers and ensuring rapid, low‐cost benefit delivery. Third, regional experience—such as Sri Lanka's expansion of universal food and cash relief during the pandemic—demonstrates that scaling up community‐based targeting and decentralized delivery mechanisms can bridge the gap between formal policy and local realities. In Bangladesh, strengthening the partnership between local government, NGOs, and microfinance institutions has shown promise in reaching informal workers with both financial and non‐financial support. Finally, policy innovation should prioritize the integration of digital identification and social registry systems, as highlighted by both Bangladesh's NSSS and World Bank recommendations, to improve the inclusiveness and responsiveness of future crisis interventions.

## Conclusion

This study provides a comprehensive and empirically grounded account of the impact of the COVID‐19 pandemic on informal workers in Bangladesh, revealing the depth of their financial vulnerability, the resilience strategies they adopted, and the crucial roles of social networks and social protection. Utilizing an exploratory sequential mixed methods approach, the study triangulated qualitative insights from in‐depth interviews with quantitative evidence from a large‐scale survey to derive robust and context‐sensitive conclusions.

The findings highlight that informal workers experienced a dramatic reduction in income—averaging over 50%—which severely compromised their ability to meet basic needs, including food, shelter, and healthcare. In response, they employed a range of coping strategies such as income diversification, reduced spending, and negotiated rent deferments. The study also underscores that social network—comprising family, friends, neighbors, and community‐based organizations—played a central role in enhancing resilience by offering financial, emotional, and material support. Importantly, the study confirms that although informal mechanisms offer temporary relief, their effectiveness is constrained in the absence of institutional support. Only 10% of respondents reported access to formal protection during the pandemic. This indicates a major policy vacuum. The regression analysis confirmed that formal protection and social networks have a significant impact on the resilience and recovery rate of informal workers.

This study adds to the resilience theory by showing empirically how income diversification is a key strategy that informal workers use to cope with economic shocks. This can be used to inform future research into resilience frameworks for economically vulnerable populations. This study highlights social networks’ positive impact on resilience. This study also highlights the importance of social capital in resilience models. Future research will examine the mechanisms through which social networks can increase resilience and identify the most effective types of support. This study provides empirical evidence about the impact of economic shocks, such as the COVID‐19 outbreak, on the incomes and financial security of informal workers. This contribution to the literature on socioeconomic vulnerability highlights the need for a more nuanced understanding of how different populations are affected by economic crises. The findings are in support of the theory that social protection programs can be essential to reducing the vulnerability of informal workers. The findings are in line with existing theories about social safety nets, and their role as a means to enhance economic stability and resilience. This study provides valuable insights for policymakers, practitioners, and researchers. The study provides an in‐depth understanding of the challenges faced by informal workers during the COVID‐19 outbreak and their strategies to overcome these challenges. Practical recommendations and theoretical contributions emphasize the importance of robust protection for vulnerable populations and the need for multifaceted support mechanisms.

These findings carry important implications for the design of inclusive social protection and recovery policies. Recovery strategies for informal workers should not rely solely on household coping mechanisms or formal welfare provision in isolation. Rather, policy design should strengthen existing community‐based support systems while also addressing the structural vulnerabilities associated with low‐income and insecure work. In Bangladesh and comparable contexts, effective recovery measures may require a combination of targeted cash or food assistance, accessible livelihood support, and locally grounded delivery mechanisms that recognize the hybrid nature of resilience in the informal economy.

## Limitations and Future Research Study

This study has several limitations that should be acknowledged. First, the data were collected from selected districts in northern Bangladesh, so the findings should be interpreted cautiously and not generalized uncritically to all informal workers in Bangladesh or other contexts. Second, the study relies partly on self‐reported information on income loss, coping strategies, and access to support, which may be affected by recall or social desirability bias. Third, the cross‐sectional nature of the quantitative data limits causal inference and captures recovery at a specific point in time rather than as a long‐term process. Future research could address these limitations through broader geographic coverage, longitudinal designs, and greater use of objective indicators to better understand how social networks and social protection shape resilience over time.

## Author Contributions

Conceived and designed the experiments: Md. Abdullah Al Mamun. Performed the experiments: Md. Abdullah Al Mamun, Khandakar Farid Uddin, Md. Shawan Uddin, A. N. M. Jahangir Kabir, and Md. Nazirul Islam Sarker. Analyzed and interpreted the data: Md. Abdullah Al Mamun, Khandakar Farid Uddin, Md. Shawan Uddin, A. N. M. Jahangir Kabir, and Md. Nazirul Islam Sarker. Contributed reagents, materials, analysis tools, or data: Md. Abdullah Al Mamun, Khandakar Farid Uddin, Md. Shawan Uddin, A. N. M. Jahangir Kabir, and Md. Nazirul Islam Sarker. Wrote the article and approved for submission: Md. Abdullah Al Mamun, Khandakar Farid Uddin, Md. Shawan Uddin, A. N. M. Jahangir Kabir, and Md. Nazirul Islam Sarker.

## Funding

The authors have nothing to report.

## Ethics Statement

Formal institutional review board approval was not obtained because, at the time of data collection, no formal ethics review mechanism was in place for this type of nonclinical, field‐based social science research within the relevant institutional context. Nevertheless, the study followed accepted ethical standards for human‐participant research. Participants were informed about the purpose of the study, the voluntary nature of participation, their right to refuse any question, and their right to withdraw at any time without consequence. Responses were anonymized, no personally identifying information was retained in the analytical dataset, and confidentiality was maintained throughout the research process.

## Consent

Oral informed consent was obtained from all participants before each interview and survey. Oral rather than written consent was used because many respondents had limited formal education, making a verbal consent process more appropriate and accessible in the field setting.

## Conflicts of Interest

The authors declare no conflicts of interest.

## Data Availability

The anonymized survey dataset, variable codebook, survey instrument, and semi‐structured interview guide are available from the corresponding author upon reasonable request for academic and non‐commercial research purposes. Because the study involves economically vulnerable human participants, full public deposition of raw qualitative materials and fully identifiable field records is restricted to protect confidentiality. Summary analytic procedures used in SPSS and NVivo can also be shared upon request.
